# Ready-to-use graphene-related material-added multi-grade oils: characterization and performance in car engine working conditions†

**DOI:** 10.1039/d4ra02406k

**Published:** 2024-06-11

**Authors:** Miquel Garcia Lleo, Valentina Sacchetti, Claudio Cacciola, Elena Medri, Simone Ligi, Andrea Liscio, Matteo Minelli

**Affiliations:** a Graphene XT srl Via d'Azeglio, 15 40123 Bologna Italy; b Istituto per la Microelettronica e i Microsistemi, Consiglio Nazionale delle Ricerche Via del Fosso del Cavaliere 100 00133 Roma Italy andrea.liscio@cnr.it; c Department of Civil, Chemical, Environmental and Materials Engineering (DICAM), Alma Mater Studiorum – University of Bologna (Italy) Bologna Italy matteo.minelli@unibo.it

## Abstract

The need for energy efficiency is leading to the growing use of additives to enhance the performance of oil in automotive engines. Great interest is focused on nano-additives even if to date there is still no practical use in commercial liquid lubricants. Herein, the potential of industrially scalable and low-cost graphene-related materials (GRMs) as additives to enhance the performance of oil in automotive engines is explored. The use of polyalkylmethacrylate dispersants, the most common key additives to formulate “green technology” lubricant oils liquid-processed GRM, is explored, investigating the role of the lateral size and the chemical analysis in the stability of the lubricant GRM dispersions. Showing the maximum duration of stability and a production method that avoids the use of strong oxidants, rheological tests were then focused on multilayered graphene flakes with sub-micrometre lateral size mixed in two commercial oil grades (5W-30 and 5W-40) under conditions similar to those of engine operation. The addition of such a filler increases the viscosity without affecting the Newtonian fluid behavior, while four-ball tests show a reduction in wear, indicating improved lubrication performance. Finally, preliminary bench-test on a commercial car engine showed increased power output corresponding to enhanced engine efficiency. The results clearly indicate the effective improvement in lubricating commercial oils due to GRM additives.

## Abstract 2

Development of long stable graphene-based lubricant blend based on fully commercial components. Friction, wear and rheological analysis and preliminary bench-test on a commercial car.

## Graphical abstract

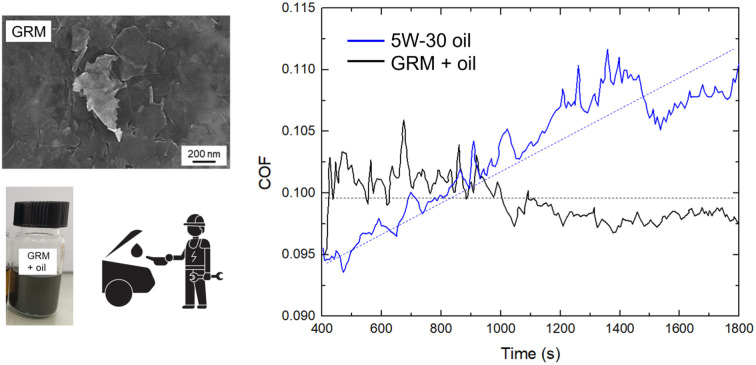

## Introduction

Research interest in the impact of transportation on global warming has surged in response to escalating concerns regarding climate change. In 2017, the transportation sector accounted for 30.8% of European energy consumption.^1^ To mitigate its effects, various strategies have been explored, including optimizing logistic networks, utilizing alternative fuels (*e.g.*, hydrogen and electric cars), and improving fuel efficiency in conventional vehicles. Friction, responsible for 4–15% of energy losses in car engines, presents a tribology-based challenge that has been investigated for years.^2^

Lubricating oils, produced from mineral or synthetic sources, are commonly employed to protect vehicle engines against wear. Most commercial oils are formulated with additives to enhance properties such as lifetime, operational stability, viscosity, and lubrication performance. Dialkyl dithiophosphate (ZDDP) is one of the most used additive, considered the best choice due to its exceptional tribological performance.^3,4^ However, the working conditions within the internal combustion engine may lead to the degradation of the additive, with the consequent release of sulfur and phosphorus by-products. It is therefore mandatory to explore alternative solutions that combine performances with chemical stability and environmental concerns.

In recent years, special attention has been paid to the analysis and development of nanomaterials as lubricant additives: their characteristic size, indeed, allows substantial reductions in the friction and wear volume, making them promising alternatives to conventional systems. These nanoadditives can be classified into three main groups depending on their chemical nature: (i) carbon allotropes and derivatives (graphene related materials – GRM^5–10^ and carbon nanotubes – CNT^11–14^), (ii) inorganic oxide nanoparticles (*i.e.*, ZnO, MgO, CuO and TiO_2_), and (iii) transition metal dichalcogenides (*e.g.*, MoS_2_).^15^ A further useful approach is to classify them based on the morphology (structure) and their number of dimensions; for example, GRM and MoS_2_ are 2D materials, CNTs are 1D, while nanoparticles can be considered as 0D at the micrometre scale.

The main research efforts have been focused on developing hybrid systems combining CNT by chemical vapor deposition (CVD) and oxide nanoparticles,^11,12,14,16–20^ leading to a significant decrease in the coefficient of friction (COF) up to 32%. Defined as the ratio between the resistive friction force (*F*_r_) by the normal force (*N*) that pushes on the objects (COF = *F*_r_/*N*), the value reduced from 0.064 for pristine oil to 0.043, showing the enhanced lubricating properties at low COF. Generally, COF for lubricating oil ranges between 0.025 and 0.4.^21,22^ However, the production scale-up of such nanomaterials still remains one of the major challenges.

GRMs have emerged as effective lubricant additives due to their low shear resistance between the layer structure, suitable size to enter the contact areas, coupled with good thermal conductivity to dissipate the heat generated during the friction process.^23–27^ Several different effective ways have been developed to ensure a stable dispersion of GRM in various base lubricants, and they can be summarized in three different strategies: (i) GRM morphological tuning (*i.e.*, size decrease, increase in surface area), (ii) GRM chemical modification, and (iii) use of dispersants.

Reducing the size of fillers is one of the most common processes to achieve uniform dispersion and to reduce the problem of particulates that can block filters.^28^ However, GRMs can aggregate to form mesoporous micro-sized structures with a large surface area by increasing the interaction with the oil. Dou *et al.* reported novel crumpled graphene balls as effective lubricant additives in poly(α-olefin) lubricant oil.^29^ Despite their compact appearance, such GRM have a great deal of free volume and solvent-accessible surface area inside, making them an effective absorber of oil, which is released upon compression, ensuring the uninterrupted wetting of the contact area.

Chemical modification is a further approach to improve the dispersivity of GRM by tuning the interaction between the filler and the oil. Lin *et al.* made use of stearic and oleic acids to graft alkylene chains onto graphene platelets *via* a cyclic heating technique to enhance the dispersion stability of GRM within the base oil employed, leading to only a minimal precipitation after 3 hours.^30^ Furthermore, COF of the base oil with 0.075 wt% modified graphene gave 0.12, showing a decrease of about 1/3 compared to that of the pure base oil.

Graphene oxide deserves a special and more detailed mention (GO), being the most common chemically-modified GRM. GO is purely a 2D material, one-atom thick, and it consists of a patchwork of graphene lattice comprising oxygen-containing defects,^31^ allowing high processability and stability in green solvent suspensions as well as making the material an ideal platform for supramolecular and covalent chemical functionalization.^32,33^ GO is attracting growing increasing interest both from customers and companies in a wide range of applicative fields, including energy storage, corrosion protection, speaker membranes, water filtration and advanced material composites, with an expected compound annual growth rate (CAGR) of 46% projected from 2022 to 2030.^34^ On adding to commercial SAE 20W-50 engine oil, a COF value of 0.057 was reached.^35^

The use of dispersants is one of the most widely used strategies in automotive lubricants with the main function of keeping the equipment clean. Moreover, in addition to preventing sludge, deposits, and varnishes, dispersants can also be used to tune the coefficients of friction, extreme pressure, and anti-wear performance in transmission fluids.^36^ The use of additives can be an effective approach that does not use hazardous chemicals and avoiding issues relating to their disposal, which can be expensive and not very eco-friendly. In the case of GRM, the use of a dispersant also allows the improvement of the dispersion stability in the case of slightly oxidized materials and, in principle, for the overall class of carbon-based materials, such as micrographites and graphene flakes (GF).^37^ Such systems are thus layered materials, not purely 2D, with thicknesses that can reach up to 20–30 nm in the case of GF, and a corresponding interface area with the lubricant lower than that of GO.

Common bottlenecks for the commercial use of GRM and other nanomaterials are related to environmental aspects and the cost, which is still high for the mass-market. Such issues are often correlated. This is a highly limiting aspect for GO in particular as its most effective production is based on functional oxidation (Hummers' methods)^6,38–40^ that makes use of strong acids and oxidants with the possible release of toxic gases and contamination of wastewater formed during the process of synthesis and purification of the material.^41^ Such methods still represent one of the main factors limiting the large-scale use of GO, although newer processes have been proved to be safer, more environment-friendly and more scalable.^42,43^ Conversely, micrographites and GF are typically produced by the mechanical exfoliation of graphite, either powder milling, grinding or in liquid phase, industrially scalable techniques that may not require severe chemical processing.^44–46^ Conventional lubricants are subject to a rather high price sensitivity (1–2 USD per L), and the lubricant additives cost about 2–3 USD per kg.^47^ Currently, the cost of industrial grade GF ranges within 50–75 USD per kg for commercial volumes (tonnage) and up to 15 USD per kg for small quantities, while GO is produced with the most cost-effective methods at 2000 USD per kg.^48–50^ However, the price pressure could be so high for mass markets, and two main aspects encourage the use of GRMs, or at least some of them: (i) the additive loading can be reduced through the use of GRM (typical loadings are between 0.5 and 30%) and (ii) GRM prices over the years have dropped rapidly and research progress on new and cheaper methods to produce such materials emerges every year due to the increase in market demand. A further important aspect to be considered, not addressed in this article, is related to the systemic cost balance (*e.g.*, lower total maintenance and/or energy costs), such as higher performance and a more durable lubricant, for example, which needs to be changed less often, allowing for a longer life price.

Herein, we studied blends based on all commercial components: GRM powders suspended in 5W-30 and 5W-40 oil using polyalkylmethacrylates (PAMA), one of the key additives to formulate lubricant oils, particularly in the area of ‘green technology’-based additives. The use of a dispersant is crucial as GRMs do not suspend in such oils. In the first part, we compare GRM powders characterized by different morphologies and chemical properties and produced by different methods. The GRMs are mixed with commercial oil suspensions with dispersants, and their physical morphology and particle sedimentation stability are studied. Thickness and lateral size are monitored by scanning electron microscopy (SEM) and atomic force microscopy (AFM) techniques. The combined use of both techniques allows to investigate thousands of nano-materials and the direct measurement of nanoflake thicknesses. Such dimensional characteristics of graphene and GO nanosheets may be crucial for their effect on the resulting macroscopic properties of the oil–GRM mixture. Moreover, the elemental analysis is provided by X-ray Photoelectron Spectroscopy (XPS), as described in detail.^31^ The stability of the GRM in oil over time is studied through sedimentation and filter agglomeration. In the following part, due to the applicability point of view of this work, the GRM–oil systems, which were stable from an industrial point of view, were selected, and tribology and wear protection were studied. Viscosity tests were performed to assess the tribological changes obtained by the addition of GRM. The wear prevention mediated by oil lubrication is evaluated using the four-ball method. Based on the comprehensive tribological characterization, a specific formulation of GRM–oil has been chosen to investigate its thermal properties and power output performance. This selection is based on its notable feature of combining long-term stability and exhibiting minimal viscosity variation as compared to the original specifications set by the manufacturer. The thermal conductivity impact of GRMs was quantified by the transient hot wire (THW) method. Lastly, preliminary bench-test and engine performance test were performed using a commercial car.

## Results and discussion

### GRM analysis and stability in nanolubricants

Commercial GRM powders produced top-down chemical or physical approaches, such as liquid phase exfoliation, thermal expansion and mechanical exfoliation, which often show a heterogeneous dispersion of the flakes with different lateral size ranging to few nm up to tens of microns, thickness from single atom thick to tens on nanometer, and the presence of defects and oxidized groups improves their dispersion in solvents.^51^ We analyzed commercial GRM powders such as graphene flakes XGS-C produced by XG-Science and IND25 purchased from Graphene-XT (IND25) and graphene oxide (marked as GO1) produced by Graphenea. The latter was also sonicated to tune the lateral dimensions and marked as GO2. The graphene flakes are all-carbon material (oxygen content < 1 wt%), while GO nanosheets show C/O ratio ≈ 1.5 (oxygen content ≈ 40 wt%). The representative SEM images of the four materials are illustrated in Fig. S1.† At the mesoscopic scale, all the four GRM show quite a large aspect ratio (lateral size/thickness > 100) with maximum thicknesses of 20 nm. Such value is at least one order of magnitude lower than the typical roughness of the engine surfaces and as well as of the instrumentation used for tribological tests. Thus, in the first approximation, we can assume that the studied GRM are deposited planarly on such surfaces.

Size and height analysis was performed using percentiles: the average size *D*_50_ is defined as the median of the sample and the size range between *D*_10_ and *D*_90_, therefore including the most representative 80% fraction of the sample. The corresponding statistical height distribution analysis is reported in Fig. S2† (more details are reported in a previous work).^52^ All the distributions are reported in Fig. S3.† All the results are summarized in Table 1. XGS-C shows quite a broad size distribution (*D*_50_ = 14 μm in the range within 6 and 38 μm), with average thickness *D*_50_ = 7 nm (corresponding to a stack of 18 single nanosheets) in the range between 5 nm and 12 nm.

**Table 1:** Physical properties of GRM and stability in oil : dispersant : GRM [95 : 4.5 : 0.5 wt%] suspension

Sample	Lateral size (μm)	Thickness (nm)	Oxygen (wt%)	Stability after 9 months
XGS-C	6; 14; 38	5; 7; 12	<1	Not stable/partial precipitation
IND25	0.1; 0.3; 0.8	4; 10; 19	<1	Stable/no sedimentation
GO1	5; 20; 55	1	40	Not stable/partial precipitation
GO2	0.1; 0.3; 0.7	1	40	Stable/no sedimentation

**Fig. 1:**
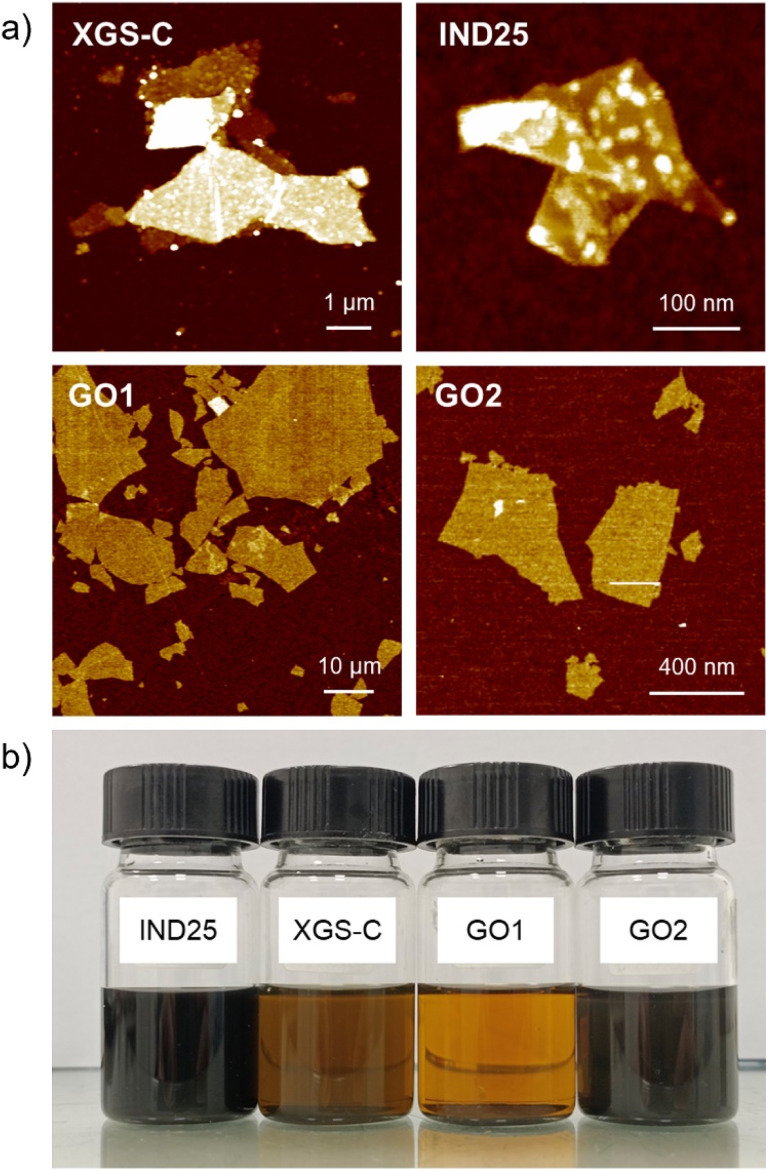
(a) AFM images of the four GRMs on silicon substrate. *Z*-Range: (XGS-C) 50 nm, (IND25) 20 nm, (GO1) 2 nm and (GO2) 2 nm. (b) Picture of the 5W-30 oil : dispersant : GRM blend suspension after 9 months of preservation in air at room temperature (20 °C) and atmospheric pressure.

Such results agree with those already reported in a previous work.^53^ The aspect ratio is larger than 10^3^. Differently, IND25 shows a lower aspect ratio (*ca.* 2 × 10^2^) with sub-micrometric lateral size (*D*_50_ = 300 nm in the range within 50 and 800 nm), suffering typical flake fragmentation with a thickness ranging between 4 nm and 19 nm (corresponding to *ca.* 10–48 layers). Differently, GO is 1 nm thick while the size is tuned in water suspension *via* sonication for 5 min and 2 hours, respectively, and after deposited on a flat substrate (Fig. 1). Increasing the sonication time, the average lateral dimension decreases from 20 μm to 300 nm and the distribution shrinks as well, *e.g.*, the large nanosheets (*D*_90_) pass from 55 μm to 0.7 μm, as previously reported.^54^

All the four GRMs are dispersed in polyalkylmethacrylates (PAMA) and then admixed with commercial oil 5W-30 and 5W-40. The choice of the relative concentrations of the three components must take into account that we are using commercial products as well as their cost effectiveness. PAMA is commonly used to increase the viscosity, and the use of large quantities may compromise the performance of the additivated nanolubricant, the actual cost of which mainly depends on the quantity of GRM added. In the case of SAE 5W oils, the quantity of PAMA does not exceed 10 wt% of the total weight.^55^

Thus, we fixed the content of oil at 95 wt% and increased the amount of IND25 from 0.125 to 1.100 wt%, monitoring a suspension diluted in base oil 293.5 : 1 by UV-vis spectroscopy at *λ* = 550 nm for 3 months. By adding GRM content up to 0.5 wt% of the total, the amount that remains in the suspension is greater than 99%. Higher concentrations of the GRM filler lead to a significant increase of the precipitated solid fraction. Then, we decided to work under conditions that maximize the quantities of graphene without appreciable residues (<1 wt%), corresponding to the oil : PAMA : GRM blend with relative concentrations (95 : 4.5 : 0.5 wt%). Oil suspensions were dispensed into 20 mL vials, which were then sealed with regular plastic caps and stored under ambient room conditions for nine months to investigate the potential precipitation of agglomerates. The vials were consistently maintained at an average temperature of 20 °C, exposed to atmospheric air conditions without any preservation using Ar or N_2_, aiming to simulate warehouse storage conditions. A first evaluation is made by visual observation as the precipitation of the flakes leads to an appreciable increase in the transparency of the GRM-added nanolubricants. As depicted in Fig. 1b, no sedimentation was observed after 9 months using IND25 and GO2 as additive in 5W-30 oil, unlike XGS-C and GO1, for which instead an appreciable sedimentation is observed, as confirmed filtering the suspensions with a commercial 7 μm filter used in engines oil recirculation (Bosch OF93, F026408893). Only, IND25 and GO2 present a complete absence of any agglomerates deposited on the filter. Similar results are observed in the case of 5W-40 lubricant, as reported in Fig. S4.†

Relevantly, only sub-micrometer GRMs form stable suspension, regardless of their chemical properties, and the defectiveness of GRMs also does not appear to play an effective role. Such results strongly indicate that the lateral size of the GRM additive affects the stability of the suspension either dynamic or static. Larger nanoflakes (XGS-C and GO1) form micrometre agglomerates that tend to block the micrometer pores of the filter. Moreover, sub-micron lateral size and large aspect ratio of the nanoflakes prove advantageous in the context of lubricant application as it enhances both the storage stability of the GRM–oil suspension and mitigates the possibility of filter blockage during recirculation. Therefore, IND25 results are considered as the most suitable candidate in the envisaged application as (i) it forms a stable dispersion in commercial oil comparable to the expected life of the lubricant mixture, and (ii) it is produced using a scalable process avoiding the use of strong oxidants.

### Viscosity test (rheology)

**Fig. 2:**
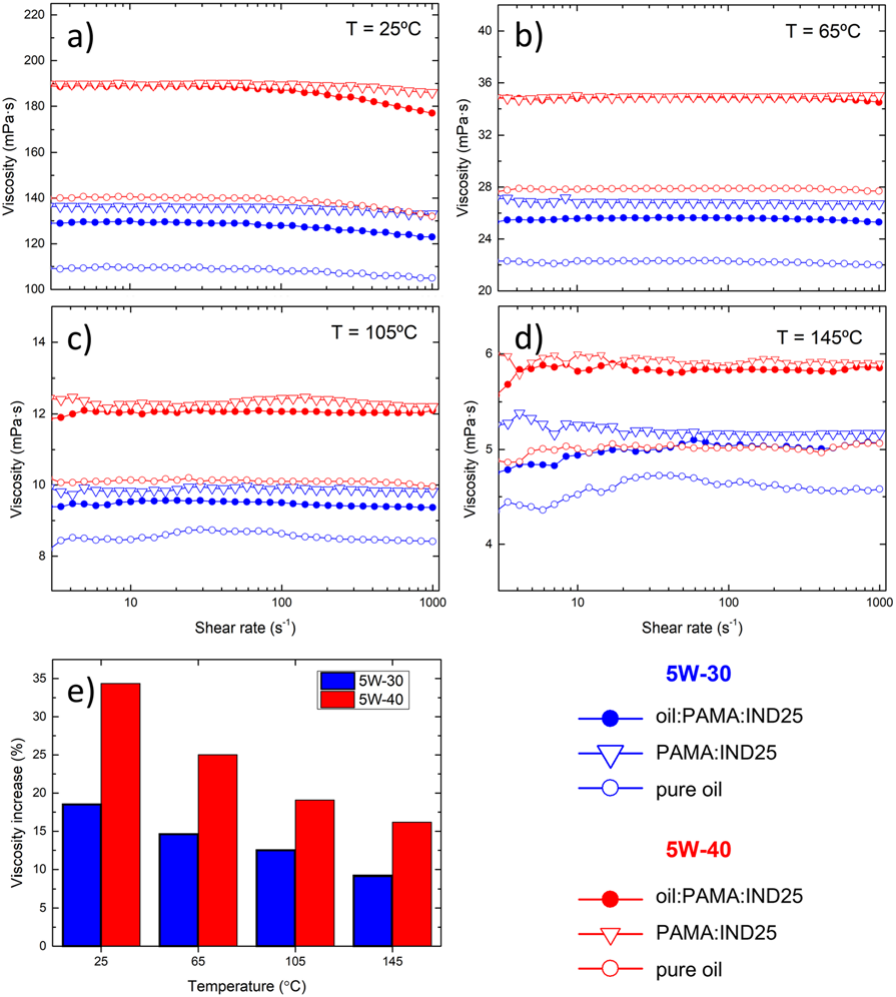
Lubricant viscosity as a function of the shear rate measured at different temperatures: (a) 25 °C, (b) 65 °C, (c) 105 °C and (d) 145 °C. Pristine oils (open circle), oil : PAMA (triangle) and oil : PAMA : IND25 (filled circle) for both 5W-30 (blue) and 5W-40 oils (red). (e) Corresponding variation of the mean viscosity values at different temperatures.

We investigate the rheological properties of the blend comparing two commercial oils (5W-30 and 5W-40) additivated by GRMs and dispersant using the relative concentrations discussed above, under conditions similar to those of engine operation. Using a rotational rheometer, we monitor the blend viscosity as function of shear rate (range 1–1000 s^−1^) and temperature from 25 to 145 °C, aiming to span from room conditions to those related to the application. Fig. 2a–d show the measurements performed with: 5W-30 (blue) and 5W-40 (red), comparing the behavior of bare oil (open circle), after adding PAMA (open triangle) and the final blends (filled circle). All curves show that the lubricants can all be considered as Newtonian fluids and the slight deviations observed at small shear rates may be considered within the experimental uncertainty due to the low viscosity measured. As expected, viscosity increases adding of PAMA dispersant and decreases as the temperature increases, following an exponential trend with the temperature reciprocal.^56^ The addition of IND25 leads to a slight decrease in viscosity (<5%) with respect to oil : PAMA. Such an effect is typically ascribed to the self-assembly of GRM flakes.^56^

In general, the viscosity of the blend is always higher than the viscosity of the pure oil. In the temperature range between 25 °C and 145 °C, the increase ranges from 35% to 15% in the case of 5W-40 and from about 20% to 10% in the case of 5W-30, as depicted in Fig. 2e. In all the cases, such values lie within the commercial viscosity range for engine lubricating oils.^57^ For instance, the kinematic viscosity range for 5W-30 and 5W-40 at 100 °C are 7.45 to 10.01 and 10.01 to 13.11 mPa s, respectively. Therefore, both oils present suitable viscosities inside the SAE standard framework. The measured viscosity follows the well-known exponential behavior with the temperature reciprocal for all the oils inspected, thus allowing us to readily evaluate the values at any temperature.

### Friction and wear tests

We investigated the anti-wear properties of lubricating oils by measuring the wear scar diameter (WSD) after the four-ball test. Normally, the smaller the wear scar diameter, the better the lubricity of the oil lubricant (see Fig. S5 and Table S1†).

**Fig. 3:**
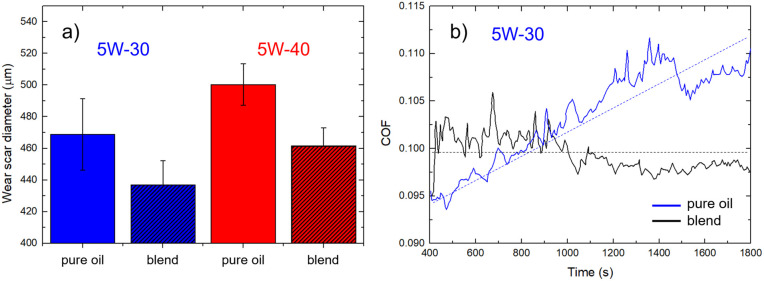
(a) Wear scar diameters (*n* = 5) resulting from the 4-ball test method comparing pure 5W-30 and 5W-40 oils with the corresponding oil : PAMA : IND25 blends. Mean values and error bars are calculated by the statistical analysis of five independent measurements. (b) Coefficient of friction (COF) of pure 5W-30 oil and the corresponding blend. Dashed lines are guide to the eyes.

Fig. 3a depicts the WSD measured on pure 5W-30 and 5W-40 oils and the corresponding blends. In general, the 5W-30-based lubricants show lower WSD values. In the case of pure oils, the WSD amount were measured to be 470 ± 20 μm and 500 ± 10 μm for 5W-30 and 5W-40, respectively. Similarly, in the case of the corresponding blends, blends the measured WSD decreases to 440 ± 20 μm and 460 ± 10 μm, corresponding to relative variations of 6–8%, without significant differences between the two oil blends in scratch reduction. The decrease in the wear clearly indicates that the addition of IND25 to the oil provides a more effective lubricating barrier in mechanical coupling. Notably, the lowest WSD is achieved in the less viscous oil blend (5W-30). Such experimental finding is interesting because it allows to mitigate the heat generation, which is typically related to viscosity. Typically, oils with lower viscosity values reduce heat generation, allowing better performance compared to more viscous oils. For this reason, we consider the 5W-30 based blend, which shows suitable tribological performance and viscosity values, for further analysis and a preliminary bench-scale test.

The change in the viscosity of a lubricant can significantly affects its tribological properties. Fig. 3b reports the behavior of the coefficient of friction (COF) at the steady-state condition of pure 5W-30 oil (blue line) and the corresponding blend (black line). We did not observe significant changes in the absolute value of the COF, which amounts to *ca.* 0.1. The main difference concerns the better friction stability of the blend as the overall COF is stable at 0.100 ± 0.002 in 1800 s of flow time, while in the case of pure oil, the COF increases roughly linearly from 0.095 to 0.110. In general, the measured curves exhibit slight variations, likely due to the four balls' vigorous spinning during the friction process. The main lubrication mechanism of graphene is derived from the formation of a tribo-film, which acts as a protective layer between the metal pairs; thus, the COF does not increase over time.^58^

### Transient hot wire method (thermal conductivity)

**Fig. 4:**
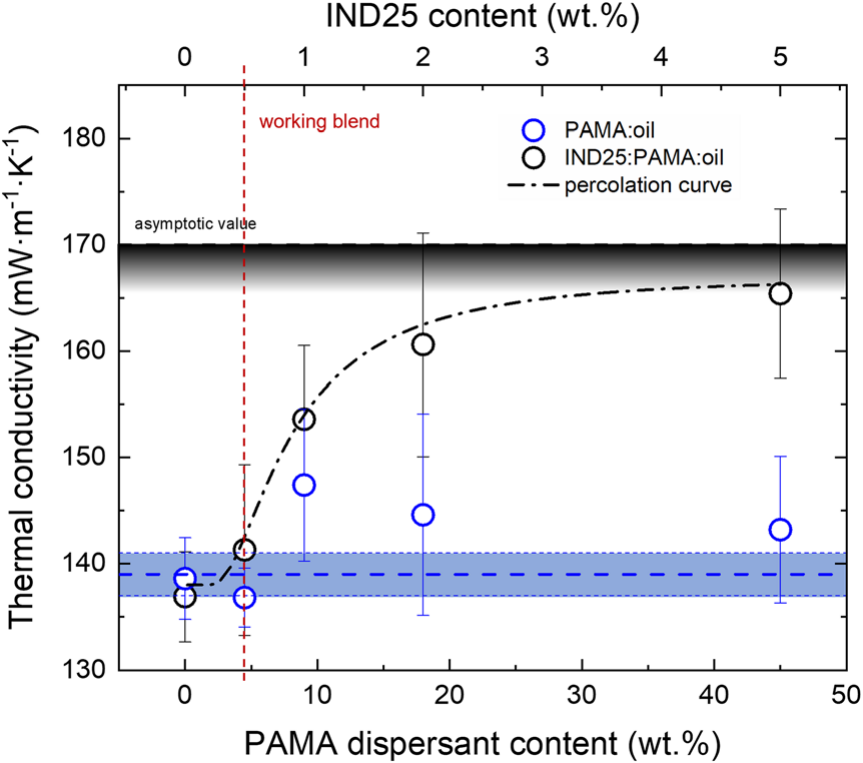
Thermal conductivity of the composite lubricant oil (5W-30) as a function of IND25 graphene platelets and PAMA-dispersant content. PAMA : oil data (blue dots) scatter around an average value (dash blue line); the shadowed area width corresponds to twice the standard deviation. Oil : PAMA : IND25 data (black dots) are fitted using a percolation threshold model curve (dash-dot black curve).^60^ Vertical red line indicates the working blend conditions.

A further relevant issue for the use of lubricating fluids is related to their ability of dissipating heat, *i.e.*, their thermal conductivity. Therefore, we explore the effect of GRMs and the dispersant in the lubricants on the thermal conductivity. The PAMA additive and IND25 (ratio 9 : 1) are dispersed in the base 5W-30 lubricant in increasing quantities for a more thorough understanding. The *κ* value obtained for the base oil was 138 ± 1 mW m^−1^ K^−1^, while no significant differences were observed on adding PAMA up to 45 wt%, as the *κ* value remained constantly equal to 139 ± 2 mW m^−1^ K^−1^ (Fig. 4). The mean and error bar values were calculated by a set of 10 independent measurements. Since the thermal conductivity of graphene flakes was 3–4 orders of magnitude larger than that of oil (*e.g.*, *κ* ≈ 10^3^ in 20 layers thick graphene flake^59^), adding very few % of GRM can strongly affect the thermal property of the blend.

Thus, we monitored the thermal conductivity of the oil : PAMA : IND25 at *T* = 20 °C, varying the IND25 quantity from 0.5 to 5 wt%, and fixing the blend PAMA : IND25 ratio as 9 : 1, as discussed above. The measured *κ* values increase with the IND25 content, forming a S-shape trend, in good agreement with the percolation threshold models commonly observed in graphite : polymer composites.^60^ Flex point is achieved at 1 wt% of IND25 and the asymptotic *κ* value tends to *ca.* 170 mW m^−1^ K^−1^ corresponding to a 20% increase with respect to the bare lubricant. It is noteworthy to highlight that such an increase in the conductivity, although significant, is much lower than the values of the single filler, suggesting the capping role of PAMA that prevents a direct percolation process between the individual IND25 flakes. Although the increase in thermal conductivity is achieved at high concentrations of the filler, the working blend (95 : 4.5 : 0.5 wt%, vertical dash red line in Fig. 4) does not show a statistically relevant thermal conductivity difference. Such experimental evidence suggests that the observed improvement in the lubricating properties cannot be attributed to improved thermal dissipation.

### Preliminary bench-scale testing

**Fig. 5:**
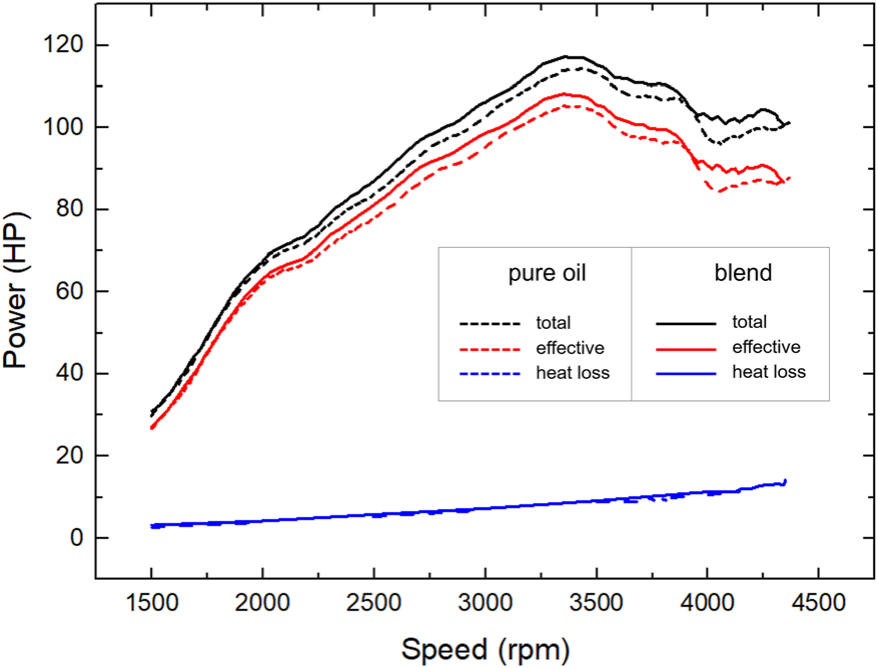
Power distributions as function of the rotational speed of pure 5W-30 oil (dashed lines) and 5W-30 : PAMA : IND25 blend (continuous lines). Achieved curves: achieved total engine power (black lines), the effective power transmitted to the wheels (red lines) and the heat losses (blue lines).

Finally, we investigated the power enhancement in a more realistic condition by means of a commercial car (Volkswagen 5P 1.6 TDI 115HP). Tests were performed comparing 5W-30 oil and the corresponding blend oil : PAMA : IND25 characterized above, diluting 250 mL of the blend in 5 L of 5W-30 oil. Fig. 5 depicts the achieved total engine power (black lines), the effective power transmitted to the wheels (red lines) and the heat losses (blue lines) for both lubricants (dash and full lines, respectively) as a function of rotational speed within the range of 1500 and 4300 rpm. Both power output curves measured using the lubricant blend were systematically larger than those obtained using pure oil. The overall increases in the power values amounted to 3 ± 1%, and the maximum power output (*P*_M_) increased from 115 ± 1 HP to 118 ± 1 HP.

Moreover, the *P*_M_ value was reached at a lower speed (3361 instead of 3420 rpm).

All the values are reported in Table 2. Differently, the heat losses curves do no present significant differences. The mechanical energy (*E*) obtained from fuel combustion may be described according to Payri *et al.*^61^ as

1 *E* = *ηQ* = *ηE*_d_*V*

where *η* is the energy conversion yield, and *ε*_gasoline_ and *V*_gasoline_ are the energy density and the volume of the gasoline, respectively.

**Table 2:** Performance parameters achieved on the bench-test for pure 5W-30 oil and the lubricant blend

Lubricant	Max power (*P*_M_) (HP)	Speed at *P*_M_ (rpm)
5W-30	115 ± 1	3420 ± 20
5W-30 : PAMA : IND25	118 ± 1	3360 ± 10

The used oil only affects the *η* parameter. Assuming the energy conversion to be proportional to the maximum power developed (*P*_M_), we obtained that the variation of the mechanical energy is only ascribed to the energy conversion yields: *P*^blend^_M_/*P*^pure oil^_M_ = *η*^blend^/*η*^pure oil^. Therefore, considering the same mechanical energy produced using pure oil and blend and considering eqn (1), we obtained the following equation.2
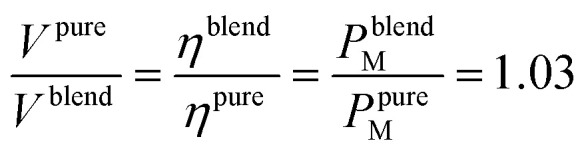


The above corresponds to 3% of fuel saving at equal mechanical performance when 5W-30 : PAMA : IND25 blend was added. Doing a rough calculation, every 30 000 km, a commercial car of the same type as the one we used needs an oil change. Considering an average fuel consumption is 5.76 USD/100 km and a gasoline oil price of 1.85 USD per L, adding IND25 : PAMA (2 USD per kg) to 5W-30 oil saved 44 USD per oil change cycle for every oil change.^62–64^

## Experimental

### GRM synthesis

Different approaches are used to produce GRM powders: liquid phase exfoliation, thermal expansion of graphite followed by physical mechanical exfoliation and modified Hummers' method, followed by the reduction of GO under N_2_ atmosphere. All methods were employed adopting the synthetic procedures already reported in previous works.^64–67^

5 g of GRM powders were added to 500 mL aqueous solution of 1 wt% sodium dodecylbenzenesulfonate, then the suspension was agitated and sonicated for 4 h (Fisher Scientific FB15047 30 W 37 kHz). After centrifugation for 30 min at 3750 rpm, the supernatant was collected and filtered through a cellulose filter. After centrifugation for 30 min at 3750 rpm, the supernatant was collected and filtered through a cellulose filter to collect XGS-C and DIN25 powders.

GO2 material was obtained by a commercial GO diluted suspension in demineralized water after sonication for 2 hours using an Elmasonic P70H ultrasonic cleaning unit.

### GRM–oil preparation

All GRM powders were then mixed with a commercial polyalkylmethacrylates PAMA from Elrois MMC in order to promote the dispersion of the nanoadditive to the lubricant oil. We tested two different commercial oils with different SAE viscosity grades (5W-30 and 5W-40). GRM and the dispersant were added to the oils with a mass ratio of 0.5 wt% and 5 wt%, respectively, while some additional blends were prepared *ad hoc* for some stability and thermal conductivity tests. All the dispersions were stirred for 30 min in order to homogenize the mixture. To improve the mixing and ensuring good lubrification performances, the oil dispersions are first processed in a three-roll machine (Morandi, Italy) before their use (one single pass).

### Characterization

The morphology of GRM powders was characterized combining scanning electron microscopy (SEM) and atomic force microscopy (AFM). SEM images were acquired using a FEI Dual Beam system (FIB-SEM) 235 with an electron beam (1 nm). AFM images were obtained in the tapping mode employing a commercial microscope (MultiMode Nanoscope 8a, Bruker). The device was equipped with a J scanner calibrated using the manufacturer's grating. Ultrasharp tips (RTESPA MPP-11120, Silicon Cantilevers, Bruker, typical force constant 40 N m^−1^, resonant frequency 300 kHz) were used. Height images were flattened to remove background slopes without the use of other filtering procedures and then analysed using Spip™ software (https://www.imagemet.com/).

The blend stability was monitored by inspecting the nanoparticle sedimentation of the dispersions 9 months after the preparation. All the lubricants were stored in room conditions and in the dark. Pristine oil : PAMA : GRM dispersions were filtered through a 7 μm standard filter to check the particle agglomeration during oil recirculation. The viscosity was measured by means a rotational Anton Paar 301e rheometer (Couette configuration) with the temperature ranging from 25 to 145 °C. A dedicated setup based on the four-ball method (ASTM D2266)^68^ was developed to study the wear properties; the main characteristics are summarized in Table S2.† We monitored the scratch diameter produced by three rotating spheres, as shown in Fig. S6,† comparing the results of a total of nine samples. The diameter of the scratch was measured by means of an optical microscope (Olympus SZ40) coupled with a digital camera. The thermal conductivity was measured using the transient hot wire method in a custom-built equipment, created based on a previously published setup.^69^ The measurement apparatus consists of a copper wire (61 μm in radius, 7 cm long) mounted on a rectangular Teflon support. The sensor was connected to a Keithley 199 multimeter for current–voltage (*I*–*V*) measurements, and a Tech Star TPR3005-2D power supply served as a constant current source. The multimeter was interfaced with a PC *via* a GPIB-USB connection, and data acquisition was controlled by LabVIEW program. Details on the optimal measurement conditions and calibration procedures are reported elsewhere.^70^

## Conclusions

In summary, we tested fully commercial nano-additivated engine lubricant based on a blend of IND25 graphene nanoplatelets, PAMA dispersant and 5W-30 engine oil. Stable for 9 months, the blend shows a better time stability of COF with respect to the pure base oil and the standard viscosity within the range given by regulation agencies. Four-ball test analysis confirmed improved wear resistance, and preliminary bench-scale tests using a commercial car showed significant increases in the power output up to 3% using the nano-additivation. The achieved results indicate that solution-processed graphene flakes IND25 are largely promising for lubrication due to the production cost and the use of an eco-friendly approach. Further analyses dedicated to the in-depth understanding of GRM-mediated mechanisms involving engine working environments are currently underway.

## Author contributions

MGL investigation; VS investigation; CC investigation; EM investigation; SL conceptualization, supervision; AL formal analysis, writing; MM conceptualization, supervision, writing.

## Conflicts of interest

There are no conflicts to declare.

## Supplementary Material

RA-014-D4RA02406K-s001

## References

[cit1] Eurostat (European Comission) , *Energy, transport and environment statistics**,* EU Publications Office, Luxembourg, LU, 2019 edn, 2019

[cit2] Wong V. W., Tung S. C. (2016). Friction.

[cit3] Barnhill W. C., Gao H., Kheireddin B., Papke B. L., Luo H., West B. H., Qu J. (2015). Front. Mech. Eng..

[cit4] Spikes H. (2004). Tribol. Lett..

[cit5] Wang W., Zhang G., Xie G. (2019). Appl. Surf. Sci..

[cit6] Paul G., Shit S., Hirani H., Kuila T., Murmu N. C. (2019). Tribol. Int..

[cit7] Mungse H. P., Kumar N., Khatri O. P. (2015). RSC Adv..

[cit8] Ali M. K. A., Xianjun H., Abdelkareem M. A. A., Gulzar M., Elsheikh A. H. (2018). Tribol. Int..

[cit9] Zheng D., Cai Z., Shen M., Li Z., Zhu M. (2016). Appl. Surf. Sci..

[cit10] Chen T., Xia Y., Jia Z., Liu Z., Zhang H. (2014). J. Nanomater..

[cit11] Kałużny J., Waligórski M., Szymański G. M., Merkisz J., Różański J., Nowicki M., Al Karawi M., Kempa K. (2020). Tribol. Int..

[cit12] Ettefaghi E., Ahmadi H., Rashidi A., Nouralishahi A., Mohtasebi S. S. (2013). Int. Commun. Heat Mass Tran..

[cit13] Ettefaghi E., Rashidi A., Ahmadi H., Mohtasebi S. S., Pourkhalil M. (2013). Int. Commun. Heat Mass Tran..

[cit14] Vardhaman B. S. A., Amarnath M., Ramkumar J., Mondal K. (2020). Mater. Chem. Phys..

[cit15] Demas N. G., Timofeeva E. V., Routbort J. L., Fenske G. R. (2012). Tribol. Lett..

[cit16] Liu K., Zhang Y., Dai F., Sun W. (2021). Powder Technol..

[cit17] Moghaddam M. A., Motahari K. (2017). Appl. Therm. Eng..

[cit18] Esfe M. H., Afrand M., Yan W.-M., Yarmand H., Toghraie D., Dahari M. (2016). Int. Commun. Heat Mass Tran..

[cit19] Esfe M. H., Rostamian H., Rejvani M., Emami M. R. S. (2018). Phys. E.

[cit20] Alirezaie A., Saedodin S., Esfe M. H., Rostamian S. H. (2017). J. Mol. Liq..

[cit21] Tortora A. M., Veeregowda D. H. (2016). Adv. Tribol..

[cit22] Baumann A., Bertsche B. (2022). Forsch. Ingenieurwes..

[cit23] Gulzar M., Masjuki H. H., Kalam M. A., Varman M., Zulkifli N. W. M., Mufti R. A., Zahid R. (2016). J. Nanopart. Res..

[cit24] Liu Y., Yu S., Shi Q., Ge X., Wang W. (2022). Lubricants.

[cit25] Ranjbarzadeh R., Chaabane R. (2021). Energies.

[cit26] Zhao J., Li Y., Wang Y., Mao J., He Y., Luo J. (2017). RSC Adv..

[cit27] Dhanola A., Gajrani K. K. (2023). J. Mol. Liq..

[cit28] Nanoparticle Technology Handbook, ed. M. Naito, T. Yokoyama, K. Hosokawa and K. Nogi, Elsevier, 3rd edn, 2018, pp. 109–168

[cit29] Dou X., Koltonow A. R., He X., Jang H. D., Wang Q., Chung Y.-W., Huang J. (2016). Proc. Natl. Acad. Sci. U. S. A..

[cit30] Lin J., Wang L., Chen G. (2011). Tribol. Lett..

[cit31] Kovtun A., Jones D., Dell'Elce S., Treossi E., Liscio A., Palermo V. (2019). Carbon.

[cit32] Melucci M., Treossi E., Ortolani L., Giambastiani G., Morandi V., Klar P., Casiraghi C., Samorì P., Palermo V. (2010). J. Mater. Chem..

[cit33] Loh K. P., Bao Q., Eda G., Chhowalla M. (2010). Nat. Chem..

[cit34] Graphene Market Size, Share & Growth Analysis Report, 2030, https://www.grandviewresearch.com/industry-analysis/graphene-industry, accessed February 7, 2023

[cit35] Kumar P., Wani M. F. (2018). Tribol. Trans..

[cit36] Tang H.-Z. and Jao T.-C., in Encyclopedia of Tribology, ed. Q. J. Wang and Y.-W. Chung, Springer US, Boston, MA, 2013, pp. 771–781

[cit37] Ota J., Hait S. K., Sastry M. I. S., Ramakumar S. S. V. (2015). RSC Adv..

[cit38] Cheng Z.-L., Li W., Wu P.-R., Liu Z. (2017). J. Alloys Compd..

[cit39] Rasheed A. K., Khalid M., Javeed A., Rashmi W., Gupta T. C. S. M., Chan A. (2016). Tribol. Int..

[cit40] La D. D., Truong T. N., Pham T. Q., Vo H. T., Tran N. T., Nguyen T. A., Nadda A. K., Nguyen T. T., Chang S. W., Chung W. J., Nguyen D. D. (2020). Nanomaterials.

[cit41] Serrano-Luján L., Víctor-Román S., Toledo C., Sanahuja-Parejo O., Mansour A. E., Abad J., Amassian A., Benito A. M., Maser W. K., Urbina A. (2019). SN Appl. Sci..

[cit42] Yu H., Zhang B., Bulin C., Li R., Xing R. (2016). Sci. Rep..

[cit43] Méndez-Lozano N., Pérez-Reynoso F., González-Gutiérrez C. (2022). Materials.

[cit44] Lee X. J., Hiew B. Y. Z., Lai K. C., Lee L. Y., Gan S., Thangalazhy-Gopakumar S., Rigby S. (2019). J. Taiwan Inst. Chem. Eng..

[cit45] Hu C.-X., Shin Y., Read O., Casiraghi C. (2021). Nanoscale.

[cit46] Beloin-Saint-Pierre D., Hischier R. (2021). Int. J. Life Cycle Assess..

[cit47] Graphene Flagship , https://graphene-flagship.eu/industrialisation/roadmap/composites-bulk-applications-and-coatings/, accessed June 2024

[cit48] Graphene Nanoplatelets for Research and Commercial Applications, https://www.ctimaterials.com/product-category/graphene-nanoplatelets/, accessed March 12, 2024

[cit49] Graphene Supermarket , https://www.graphene-supermarket.com/, accessed February 8, 2023

[cit50] Bointon T. H., Barnes M. D., Russo S., Craciun M. F. (2015). Adv. Mater..

[cit51] Backes C., Abdelkader A. M., Alonso C., Andrieux-Ledier A., Arenal R., Azpeitia J., Balakrishnan N., Banszerus L., Barjon J., Bartali R., Bellani S., Berger C., Berger R., Ortega M. M. B., Bernard C., Beton P. H., Beyer A., Bianco A., Bøggild P., Bonaccorso F., Barin G. B., Botas C., Bueno R. A., Carriazo D., Castellanos-Gomez A., Christian M., Ciesielski A., Ciuk T., Cole M. T., Coleman J., Coletti C., Crema L., Cun H., Dasler D., Fazio D. D., Díez N., Drieschner S., Duesberg G. S., Fasel R., Feng X., Fina A., Forti S., Galiotis C., Garberoglio G., García J. M., Garrido J. A., Gibertini M., Gölzhäuser A., Gómez J., Greber T., Hauke F., Hemmi A., Hernandez-Rodriguez I., Hirsch A., Hodge S. A., Huttel Y., Jepsen P. U., Jimenez I., Kaiser U., Kaplas T., Kim H., Kis A., Papagelis K., Kostarelos K., Krajewska A., Lee K., Li C., Lipsanen H., Liscio A., Lohe M. R., Loiseau A., Lombardi L., López M. F., Martin O., Martín C., Martínez L., Martin-Gago J. A., Martínez J. I., Marzari N., Mayoral Á., McManus J., Melucci M., Méndez J., Merino C., Merino P., Meyer A. P., Miniussi E., Miseikis V., Mishra N., Morandi V., Munuera C., Muñoz R., Nolan H., Ortolani L., Ott A. K., Palacio I., Palermo V., Parthenios J., Pasternak I., Patane A., Prato M., Prevost H., Prudkovskiy V., Pugno N., Rojo T., Rossi A., Ruffieux P., Samorì P., Schué L., Setijadi E., Seyller T., Speranza G., Stampfer C., Stenger I., Strupinski W., Svirko Y., Taioli S., Teo K. B. K., Testi M., Tomarchio F., Tortello M., Treossi E., Turchanin A., Vazquez E., Villaro E., Whelan P. R., Xia Z., Yakimova R., Yang S., Yazdi G. R., Yim C., Yoon D., Zhang X., Zhuang X., Colombo L., Ferrari A. C., Garcia-Hernandez M. (2020). 2D Mater..

[cit52] Liscio A. (2013). ChemPhysChem.

[cit53] Xia Z. Y., Pezzini S., Treossi E., Giambastiani G., Corticelli F., Morandi V., Zanelli A., Bellani V., Palermo V. (2013). Adv. Funct. Mater..

[cit54] Liscio A., Kouroupis-Agalou K., Betriu X. D., Kovtun A., Treossi E., Pugno N. M., De Luca G., Giorgini L., Palermo V. (2017). 2D Mater..

[cit55] Neveu C. D. , Sondjaja R., Stöhr T. and Iroff N. J., in Polymer Science: A Comprehensive Reference, ed. K. Matyjaszewski and M. Möller, Elsevier, Amsterdam, 2012, pp. 453–478

[cit56] Pakharukov Y., Shabiev F., Safargaliev R., Mavrinskii V., Vasiljev S., Ezdin B., Grigoriev B., Salihov R. (2022). J. Mol. Liq..

[cit57] SAE International Recommended Practice , Engine Oil Viscosity Classification, SAE Standard J300_202104, Revised April 2021, Issued June 1911, 10.4271/J300_202104

[cit58] Xu Y., Peng Y., Dearn K. D., Zheng X., Yao L., Hu X. (2015). Wear.

[cit59] Jang W., Chen Z., Bao W., Lau C. N., Dames C. (2010). Nano Lett..

[cit60] Sudduth R. D. (2019). J. Appl. Polym. Sci..

[cit61] Payri F., Olmeda P., Martín J., Carreño R. (2015). Appl. Therm. Eng..

[cit62] Volkswagen Golf 5-Door 1.6 TDI 115hp , Car.info, 2017, https://www.car.info/en-se/volkswagen/golf/golf-5-door-16-tdi-2017-25481634, accessed May 14, 2024

[cit63] European Fuel Prices, https://www.cargopedia.net/europe-fuel-prices, accessed May 16, 2024

[cit64] Consumi Volkswagen Golf 7 (dal 2012 al 2019) – reali indicati dai possessori, misurati dalle riviste e dichiarati dalla casa, https://www.tagliandiauto.it/it/consumi-reali/130-volkswagen/699-consumi-volkswagen-golf-reali-indicati-dai-possessori-misurati-dalle-riviste-e-dichiarati-dalla-casa, accessed May 16, 2024

[cit65] Rea R., Ligi S., Christian M., Morandi V., Baschetti M. G., De Angelis M. (2018). Polymers.

[cit66] Treossi E., Melucci M., Liscio A., Gazzano M., Samorì P., Palermo V. (2009). J. Am. Chem. Soc..

[cit67] World Intellectual Property Organization , WO Pat., WO2014/033274, 2012

[cit68] D02 Committee , Test Method for Wear Preventive Characteristics of Lubricating Fluid (Four-Ball Method), ASTM International

[cit69] Azarfar S., Movahedirad S., Sarbanha A. A., Norouzbeigi R., Beigzadeh B. (2016). Appl. Therm. Eng..

[cit70] Khayet M., Zárate J. M. O. D. (2005). Int. J. Thermophys..

