# Supplementary material for: Ready-to-use graphene-related material-added multi-grade oils: characterization and performance in car engine working conditions
Source: RSC Adv. 2024 Jun 11;14(26):18730–8. doi: 10.1039/d4ra02406k (PMC11166019; doi:10.1039/d4ra02406k)
Supplement: RA-014-D4RA02406K-s001 [file RA-014-D4RA02406K-s001.pdf]

Electronic Supplementary Material (ESI) for RSC Advances.
This journal is © The Royal Society of Chemistry 2024

Electronic Supporting Information

Ready-to-use Graphene Related Materials added multi-grade oils: Characterization and performance in car engine working conditions

Miquel Garcia Lleo, Valentina Sacchetti, Claudio Cacciola, Elena Medri, Simone Ligi, Andrea Liscio, Matteo Minelli

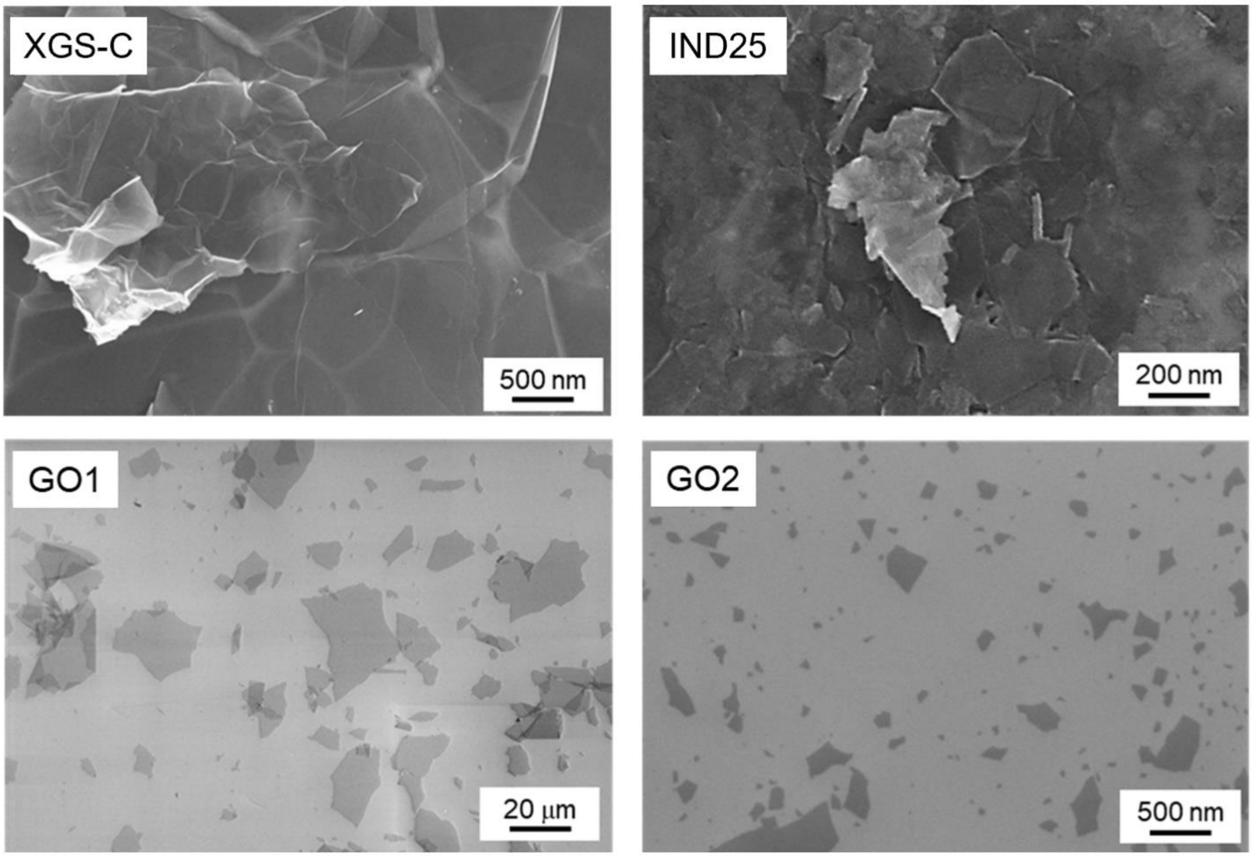

Figure S1. SEM images of GRM samples deposited on silicon wafers.

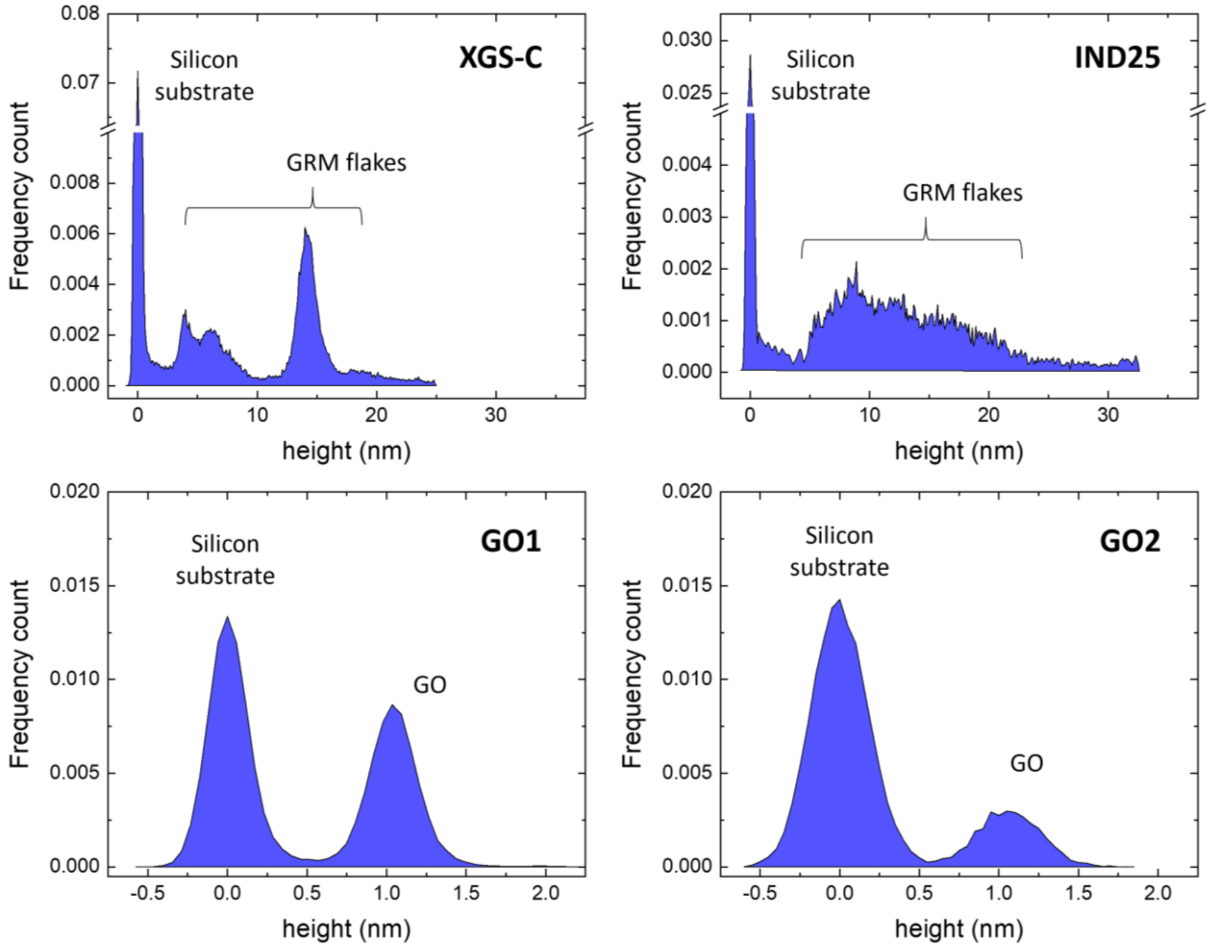

Figure S2. Height histogram distribution of the corresponding AFM images reported in the figure 1 of the main text.

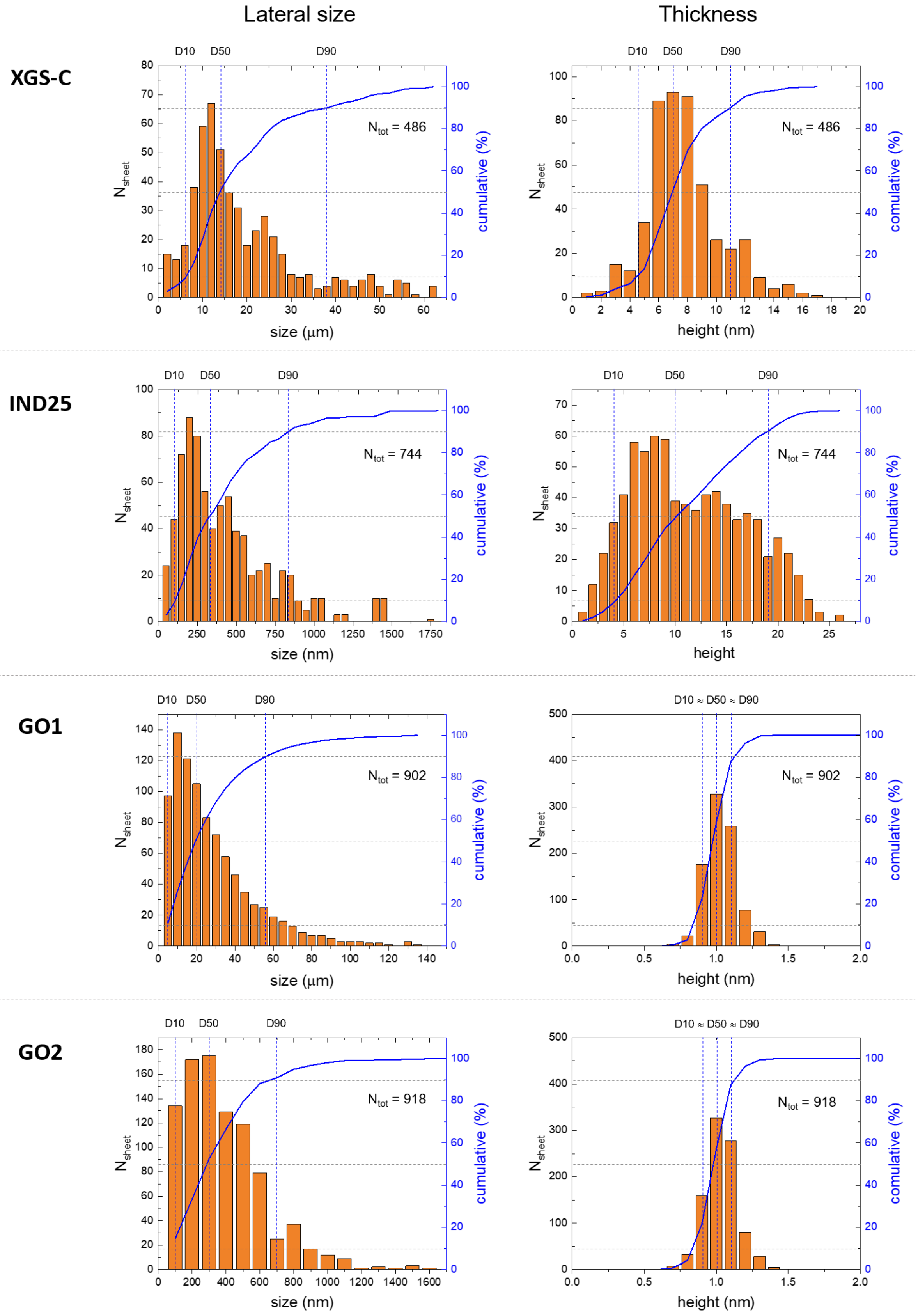

Figure S3. Lateral size and height distributions of the four GRMs (orange bar), corresponding cumulative curves (blue lines) and percentiles.

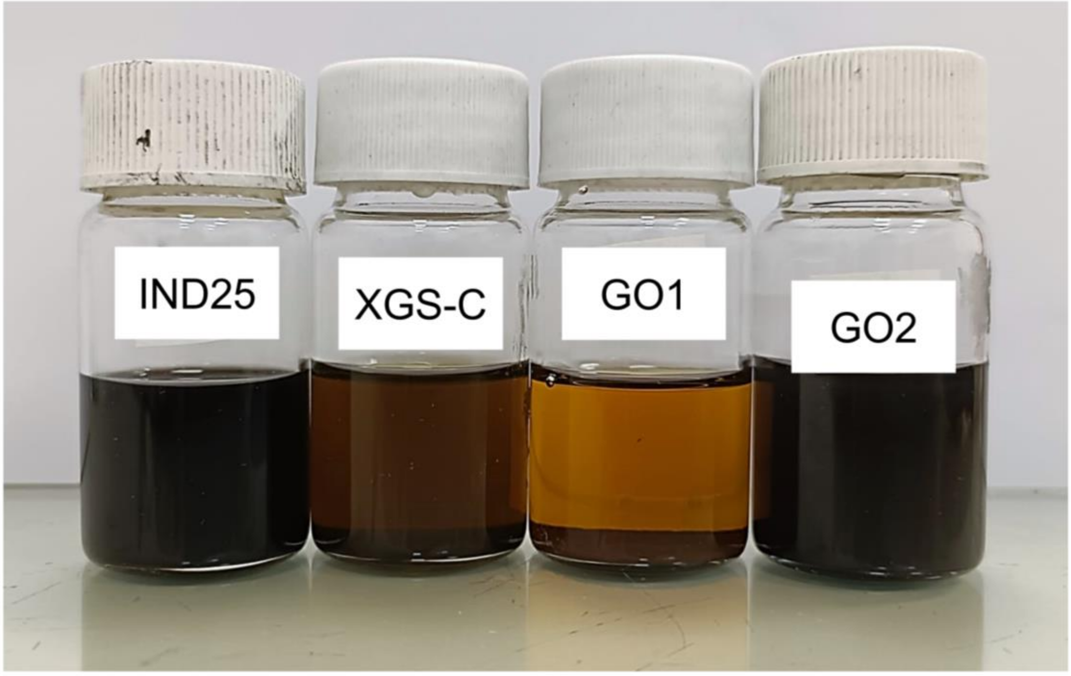

Figure S4. Picture of the 5W-40 oil:dispersant:GRM blend suspension after 9 months of preservation.

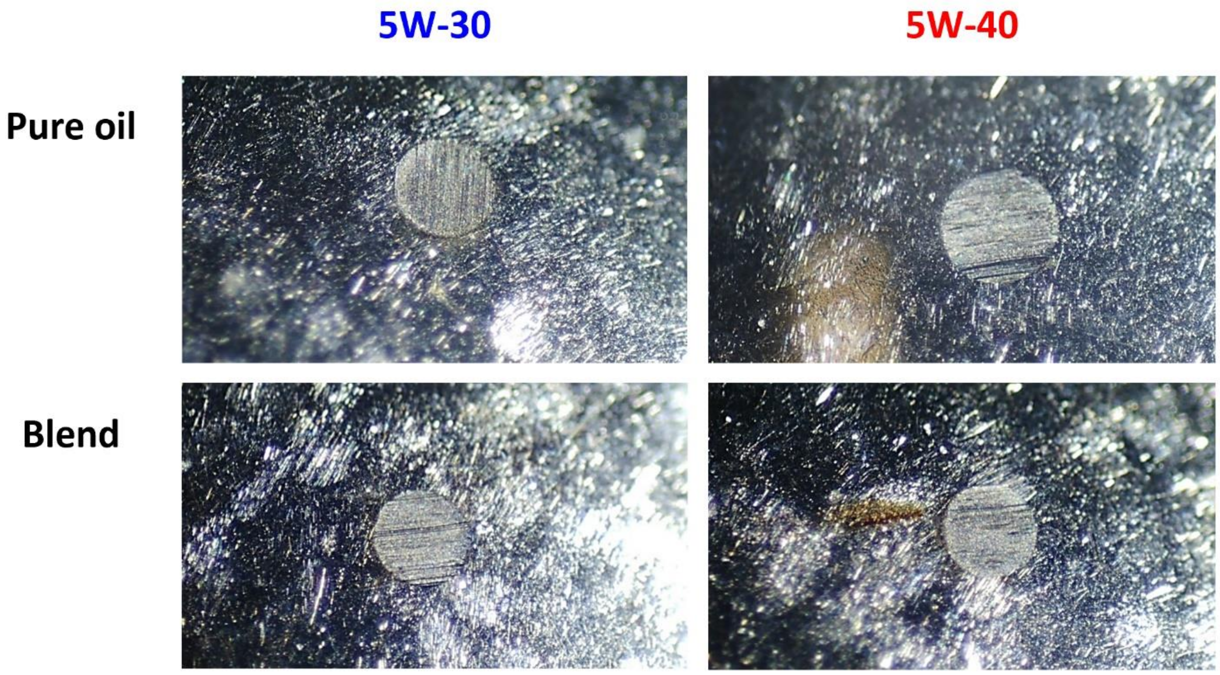

Figure S5. Optical images of wear diameter scar after four ball tests.

Table S1. Measured wear diameter scar

	5W-30	5W-40
Pure oil	470±20 μm	500±10 μm
Blend	440±20 μm	460±10 μm

Table S2. Experimental conditions of 4-ball test setup in comparison with ASTM D2266 method

	**ASTM D2266**	**4 BALL setupP**
**Ball diameter**	12.7 mm	14 mm
**Steel**	AISI E-52100	AISI E-52100
**Temperature**	75 °C	65-85
**Rotation speed**	1200±60 rpm	1850±10 rpm
**Load**	147 N or 392 N	345 N
**Time**	60 minutes	40 minutes

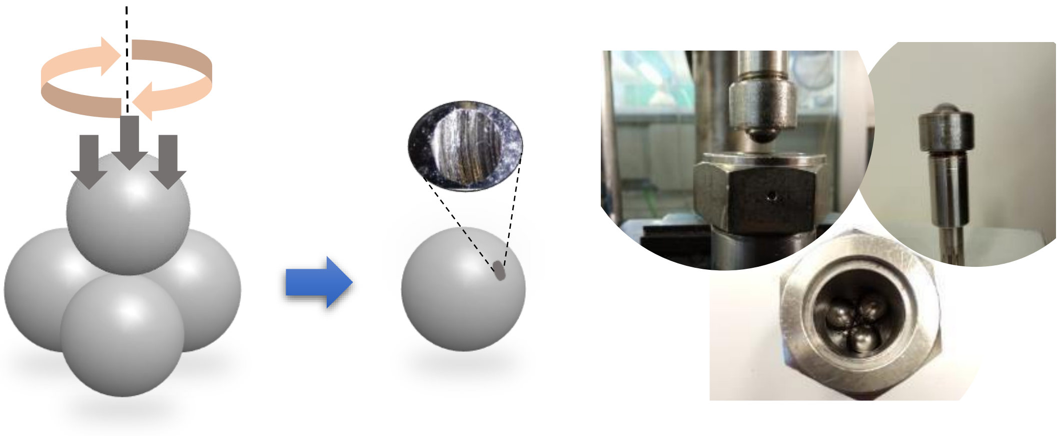

Figure S6. Scheme and pictures of the four ball test setup.
